# Effectiveness and safety of light vegetarian diet and Qingjiang Tiaochang Recipe for functional constipation: An exploratory study protocol for randomized controlled trial

**DOI:** 10.1097/MD.0000000000021363

**Published:** 2020-09-25

**Authors:** Xinyuan Liu, Yu Liu, Jialiang Chen, Huijing Wang, Qianqian Wang, Zuohu Niu, Zhangjun Yun, Bingzhi Ma, Shunkun Yao

**Affiliations:** a School of Graduates, Beijing University of Chinese Medicine; b Department of Gastroenterology of Traditional Chinese Medicine, China-Japan Friendship Hospital; c Peking University China-Japan Friendship School of Clinical Medicine, Peking University; d Department of Pharmacy, China-Japan Friendship Hospital, Beijing, China.

**Keywords:** clinical trials, functional constipation, herbal medicine, RCT, vegetarian

## Abstract

**Introduction:**

Functional constipation is a chronic disease that is common in children and adults around the world. The treatments for functional constipation include diet and lifestyle interventions, medications, and surgery. The diet pattern plays an important role in the occurrence of constipation. We found in clinical practice that simple application of drugs cannot achieve long-term relief of constipation, and a large number of patients are not satisfied with the existing treatment. We have concluded that Qingjiang Tiaochang Recipe (QJTCR) and light vegetarian diet (LVD) can effectively improve constipation. However, there is no enough evidence for the description of the effect. This protocol aims at exploratorily investigating effectiveness and safety of LVD and QJTCR following a rigorous clinical trial.

**Methods and analysis:**

We will recruit 90 patients to participate in this prospective, placebo-controlled, randomized trial, and exploratory study at the China-Japan Friendship Hospital, including traditional Chinese medicine group, placebo + diet group, traditional Chinese medicine + diet group. Patients in the diet intervention group must strictly abide by LVD, and the study will continue for 28 days. During the intervention period, we need to record a designed diary to assess diet quality and defecation. The primary outcomes for this clinical study were weekly complete spontaneous bowel movements. The secondary outcomes were constipation-related symptom rating scale, traditional Chinese medicine syndrome scale, and 48-hour gastrointestinal transit time, high-resolution anorectal manometry, Bristol stool score, constipation quality of life assessment scale, constipation symptoms self-assessment scale, short-chain fatty acids in feces. In addition, the study will determine the safety of the intervention.

## Abstract 2

Supplemental Digital Content is available in the text

## 1 Introduction

Functional constipation (FC) is a common functional gastrointestinal disease and one of the most common gastrointestinal diseases of outpatients. The incidence of constipation in the world is about 15%,^[1,2]^ In China, the prevalence rate varied from 6% to 13% from 2010 to 2016,^[3]^ and the prevalence rate of constipation among the elderly in Beijing was 13%.^[3]^ FC does not threaten the life of the patient, but it seriously affects the patient's quality of life (QOL) and causes high medical expenses.^[4]^

Prolonged colonic transit time, pelvic floor muscle dysfunction, and their combined effects are recognized mechanisms for the development of FC. Gender, age, diet, education, and neurological diseases are the main influencing factors of FC.^[5]^ The occurrence of constipation is inversely related to the intake of dietary fiber (fruits, vegetables). A low-fiber diet can induce constipation. The latest diagnosis of FC is based on the criteria of Rome IV Standard, including laborious defecation, dry or lumpy stool, incomplete defecation, anorectal obstruction, prolonged defecation cycle, and manually assisted defecation. Two or more of the above items need to be included with at least 25% frequency for each one. Meanwhile, loose stool is rarely present without laxative and irritable bowel syndrome should be excluded.^[6]^

The treatment of constipation should pay attention to comprehensive management. The routine management of adult functional constipation includes lifestyle intervention, pelvic floor intervention (rectal emptying disorder), and drug treatment. Currently, medical treatment is not satisfactory in continuously improving the symptoms and quality of life of FC patients, and side effects such as electrolyte disturbance, diarrhea, and nausea are often reported.^[7]^ Therefore, it is necessary to find a safe and effective treatment. Reports show that out of 6318 Chinese constipation adults, only 25.1% choose laxatives, and 81.7% of them prefer lifestyle and eating habits as treatment options.^[8]^

Studies have confirmed that the fecal microbiome of FC patients will change, such as the reduction of the number of Preobacterium strains and the decrease of the abundance of microflora.^[9]^ These microbiome changes may be result from low-fiber diet.^[10]^ Different dietary habits, such as low intake of water, fruits and vegetables, and high intake of spicy and greasy foods, all contribute to the increased prevalence of FC. Similarly, increasing the intake of whole fruits can protect the health of the colon and gastrointestinal tract.^[3,11,12]^ The report pointed out that 57% of the intestinal flora depends on the diet structure. Five days of dietary changes may quickly change the human intestinal flora. Therefore, diet is closely related to intestinal flora.^[13]^ Diet can cause constipation by affecting the flora. For example, a lack of isoflavones can cause intestinal flora imbalance, which can lead to constipation.^[12]^ Light vegetarian diet (LVD) is a healthy eating pattern summarized in clinical work. LVD is characterized by vegetarianism, avoiding high-temperature processing, and reducing flavoring.^[14]^

Traditional Chinese medicine formula can effectively improve gastrointestinal symptoms and intestinal health of adults with digestive system diseases. The mechanism is to reduce intestinal permeability, enhance the richness of intestinal flora, and increase the number of probiotics.^[15,16]^ Chinese herbal medicine can improve various chronic diseases, such as coronary heart disease, diabetes, obesity, depression, tumors, etc., by regulating the intestinal flora.^[17]^ Qingjiang Tiaochang Recipe (QJTCF) is a Chinese medicine prescription for treating functional constipation summarized in the clinical practice. The prescription does not contain drugs that can cause melanosis of the colon, such as rhubarb and aloe.

To date, there is no rigorous evidence to support the effectiveness and safety of LVD and QJTCR; thus, this needs authentication based on clinical trials in human subjects. In this protocol, we will design a randomized controlled trial (RCT) to systematically verify that point.

## 2 Methods and design

### 2.1 Study objective and hypotheses

This exploratory research project aims to study the effectiveness of QJTCF and light vegetarian therapy for functional constipation and the changes of short-chain fatty acids in feces before and after treatment.

### 2.2 Study design

**Figure 1:**
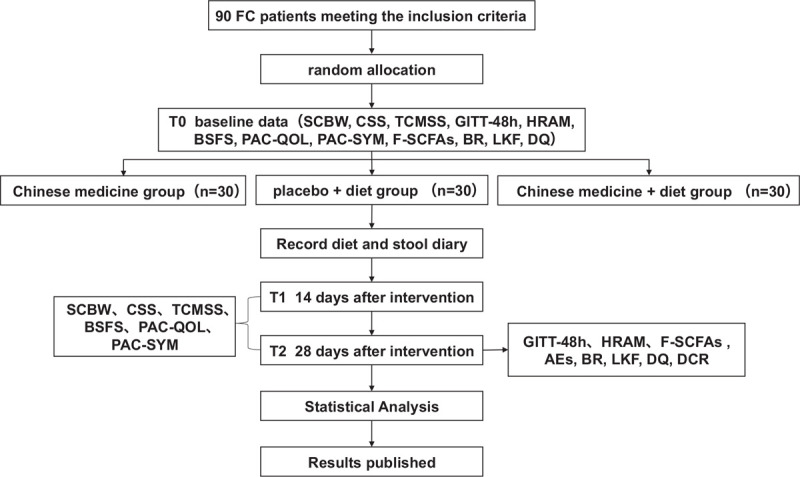
Study design. 48-h GITT = 48-hour gastrointestinal transit time, AEs = adverse events, BR = blood routine, BSFS = Bristol stool form scale, CSBM/W = complete spontaneous bowel movements per week, CSS = constipation-related symptom rating scale, DCR = dietary compliance rate, DQ = diet quality, F-SCFAs = short-chain fatty acids in feces, HRAM = high-resolution anorectal manometry, LKF = liver and kidney function, PAC-QOL = patient assessment of constipation quality of life, PAC-SYM = patient assessment of constipation symptom, TCMSS = traditional Chinese medicine syndrome scale.

**Figure 2:**
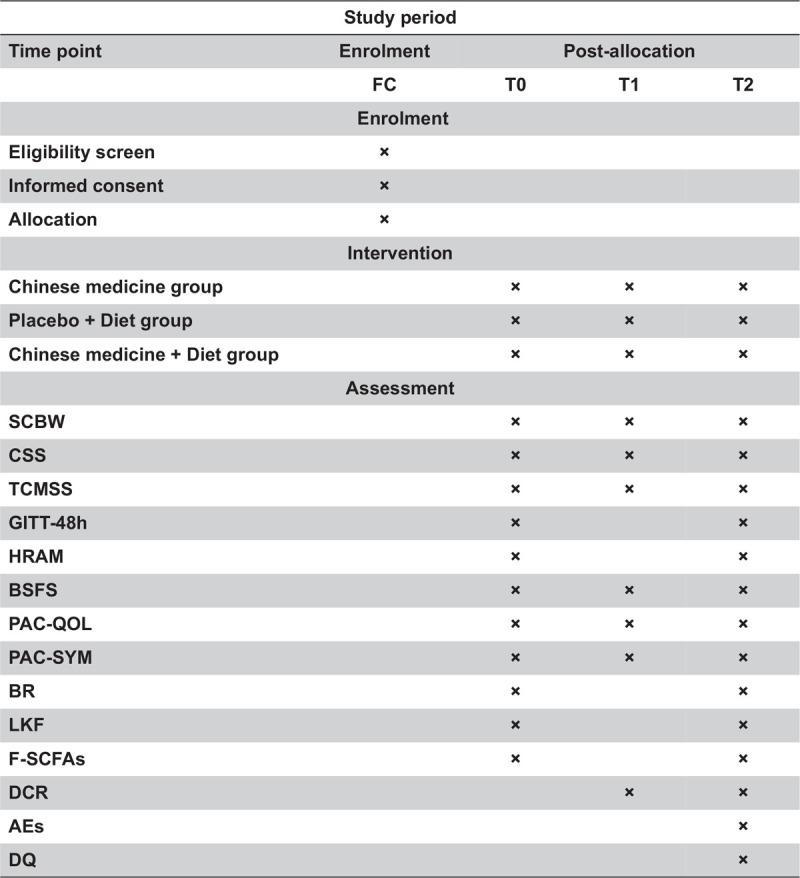
SPIRIT figure: schedule of enrolment, interventions, and assessments. 48-h GITT = 48-hour gastrointestinal transit time, AEs = adverse events, BR = blood routine, BSFS = Bristol stool form scale, CSBW = complete spontaneous bowel movements per week, CSS = constipation-related symptom rating scale, DCR = dietary compliance rate, DQ = diet quality, F-SCFAs = short-chain fatty acids in feces, HRAM = high-resolution anorectal manometry, LKF = liver and kidney function, PAC-QOL = patient assessment of constipation quality of life, PAC-SYM = patient assessment of constipation symptom, TCMSS = traditional Chinese medicine syndrome scale.

The protocol was drafted using the SPIRIT guidelines and the results will be reported following the checklist of the Consolidated Standards of Reporting Trials statement (see Supplementary Table 1). All participants will be informed of the purpose of the study and obtain their informed consent before participating in this parallel group study. According to the inclusion and exclusion criteria, a total of 90 patients with functional constipation are expected to be recruited. Then they were randomly assigned to the Chinese medicine group (group A), placebo + diet group (group B), Chinese medicine + diet group (group C) at the ratio of 1: 1: 1. The patient filled out the case report form (CRF) after confirming enrollment, and the researchers recorded the patient's symptoms, bowel movements, etc., on day 14 (phone follow-up) and day 28. Interventions were provided to subjects based on the enrollment number, but neither the patient nor the investigator knew whether the patient was receiving drug or placebo. The entire study design is shown in the figure (see Fig. 1). A SPIRIT figure for the schedule of enrolment, interventions, and assessments is also presented (see Fig. 2).

### 2.3 Exploratory study

Light vegetarian diet is a new type of dietary model, and QJTCF is a safe and effective prescription. So far, there are no randomized controlled studies on LVD and QJTCF, so this study is an exploratory study. The essence of diet therapy is cognitive behavior therapy. Chinese medicine is complementary and alternative medicine in many countries. Through this research, doctors can pay more attention to the application of cognitive behavioral therapy and traditional Chinese medicine.

### 2.4 Inclusion criteria, exclusion criteria, and exit criteria

Participants must meet inclusion criteria (see Table 1) and be excluded from exclusion criteria (see Table 1), while exit criteria (see Table 1) are set for participants who cannot complete the study. Once a patient is included in the trial, the researchers in our team will keep in touch with him or her and try to solve the problems during the treatment to reduce the loss of the intervention. During the trial, patients have the right to withdraw at any condition and will not affect subsequent treatment. The data will be recorded truthfully for further analysis.

**Table 1:**
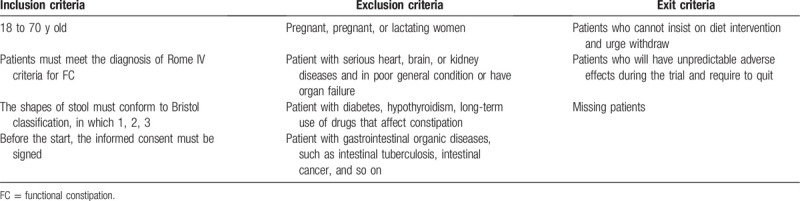
The inclusion criteria, exclusion criteria, and exit criteria for the trial.

### 2.5 Sample size

Because there is still insufficient clinical evidence, this study is a small sample exploratory study with a total sample size of 90 people (each treatment group has 30 samples).

### 2.6 Recruitment

Participants will be recruited from the outpatients and inpatients of Gastroenterology Department and traditional Chinese medicine (TCM) Gastroenterology Department in China-Japan Friendship Hospital in Beijing through roll-up banners, posters, Wechat, and the hospital official website. Two or more experts (at least 1 from Gastroenterology Department and 1 from TCM Gastroenterology Department) will decide the eligibility unanimously following the inclusion and exclusion criteria. It will be insured that both physicians and patients have no financial interests. All eligible recruiters will sign a written informed consent and those who accept the purpose, benefits, and adverse effects of the study will be enrolled.

### 2.7 Intervention

The 2 interventions are light vegetarian diet (see Table 2) and QJTCF (see Table 3). QJTCF (Tongrentang Chinese Medicine) will be made into dry Chinese medicine powder. The placebo will be composed of bitters (Zhejiang Bodanheng Food Ingredients Co, Ltd), caramel color (Guilin Hongxing Food Ingredients Co, Ltd), QJTCF powder and dextrin (Liaoning Dongyuan Pharmaceutical Co, Ltd) with 2: 3: 5: 100 made into brown powder. Use the same type of aluminum foil to pack herbal powder and placebo (12 g per pack). The time for taking Chinese medicine and placebo is half an hour after breakfast and dinner, one sachet at a time, rinsed with warm water.

**Table 2:**
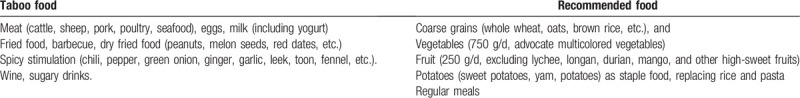
Specific content of light vegetarian food.

**Table 3:**
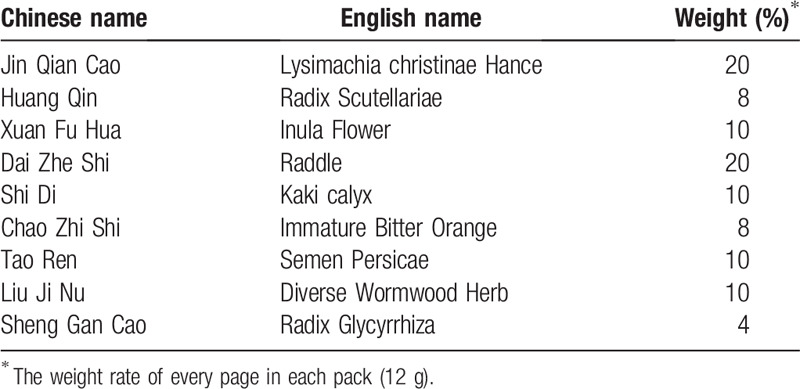
The component of Chinese herb powder.

Patients with dietary interventions should follow the above, while patients without dietary interventions will continue their previous dietary habits. The treatment period is 28 days. During the treatment period, record the daily diet and defecation diary. The diet diary will be designed using a semi-quantitative food frequency method, and the defecation diary will collect information about the feeling of defecation, the shape of feces, and the time of each bowel movement. On the 14th day, the patient will be followed up by phone to know the status of the treatment process, and calculate the average number of complete spontaneous bowel movements (CSBMs) per week for the first 14 days, the average score of constipation symptoms and TCM syndrome. After 28 days of treatment, a second follow-up visit will be conducted to understand the condition of constipation (same as the first follow-up visit), and the patient will be required to complete the examination (48 hours gastrointestinal transit Time [GITT], high-resolution anorectal manometry [HRAM], blood routine [BR], liver and kidney function [LKF]). The short-chain fatty acids of the patient 's fresh stool (<30 min) should also be checked and the diary should be retrieved. During the intervention, patients will keep in touch with the researchers. The patient's diet and stool diary will be filled out by the doctor. Keep in touch with each patient in the treatment period through mobile phone software (WeChat), ask the patient to send 3 meals to the doctor's mobile phone, ask the patient about the stool on the day at 7 o’clock every night, and urge patients in the diet intervention group to insist on light vegetarian diet.

### 2.8 Randomization, allocation concealment, and partly double-blind

Third parties not involved in the study will use Excel 2013 software (Microsoft Corporation, Redmond, WA) to generate 90 independent random numbers and divide them by 3. The rest will be 1 in group A, 2 in group A, and 0 in group C. A, B, and C are defined as Chinese medicine group, placebo + diet group, Chinese medicine + diet group. If the enrolled patients fall off, the total number of enrolled patients exceeds 90, and they will be randomly grouped again according to needs. Patient serial numbers will be generated in the order of enrollment. When distributing medicines, neither the doctor nor the patient can know what kind of medicine. The drug number information will be retained, encapsulated, and stored in an opaque envelope, and kept by a third party. When the patient is enrolled, the envelope based on their serial number will be delivered to the researcher. After the trial, the third party will expose blindness. Because dietary intervention is a set of dietary principles rather than specific foods, it is difficult to be blinded, so only drugs are double-blind to reduce information bias.

### 2.9 Emergency unblinding

In case of any serious adverse events or emergency, the researcher will report to the supervisor and the main researcher to decide whether to open the emergency letter. Once opened, the case will be treated as exfoliated, excluded from the efficacy analysis, but will remain included in the adverse reaction analysis. Details of the unblinding cause, date, treatment situation, and results will be reported in the CRF and will be signed by the administrator.

### 2.10 Quality control

Participants’ diet quality (DQ) will be assessed at the baseline and after the intervention. The foods involved are in LVD content.

### 2.11 Data collection

#### 2.11.1 Baseline information

Baseline information will include demographic data and clinical data from the participants. Demographic data includes gender, age, height, weight, nationality, occupation, and education level, etc. Clinical data includes past medical history, drug allergy history, the history of medications used to treat or induce constipation, dietary habits in the past year (see Supplementary Table 1), the primary outcome, and secondary outcomes.

#### 2.11.2 Primary outcome

The number of CSBM per week is the primary outcome. It is effective when the number before the intervention subtracted from the number after the intervention is more than or equal to 1.

#### 2.11.3 Secondary outcomes

Constipation-related symptom rating scale: The scale for constipation-related symptoms is used for investigating the degree and frequency of symptoms mentioned in Roman IV diagnostic criteria for FC. The degree of discomfort is assessed by visual analog scale, in which 0 represents no discomfort and 10 represents very strong discomfort. The final scores are obtained with the multiplication of the 2 and then divided by the average number of defecations per week (see Supplementary Table 2). Percent improvement will be calculated as follows: 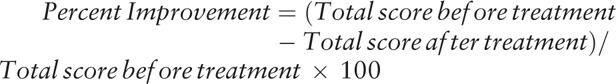


Recovery means symptoms disappeared; Marked improvement means percent improvement is more than or equal to 80%; Progress means percent improvement is more than or equal to 50% but less than 80%; Ineffective means percent improvement is less than 50%.

Traditional Chinese medicine syndrome scale (TCMSS): The TCMSS is established by collecting information about effects of relevant syndromes of TCM constipation, which are assigned 0, 1, 2, and 3 points representing normal, mild, moderate, and severe degrees, respectively. Normal refers to the absence of this syndrome, mild refers to a slight impact on life, moderate means a significant impact on life, and severe refers to the serious impact on life (see Supplementary Table 3). Nimodipine method is used for evaluating the effects of relevant syndromes of TCM constipation. 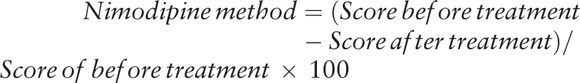


Symptoms, signs disappeared or basically disappeared and syndrome integral reduced more than or equal to 95% is considered clinical cure; Significantly effective means that symptoms, signs significantly have improved, and syndrome integral has reduced more than or equal to 70%; Effective means that symptoms, signs have improved and syndrome integral has reduced more than or equal to 30%; Ineffective means that symptoms, signs have no improved or have even aggravated and syndrome score has reduced less than 30%.

Forty-eight hours GITT: 48-hour GITT is a simple method for evaluating the function of gastrointestinal motility. The barium residue rate is the ratio of the number of barium remaining in the body 48 hours after swallowing 20 barium bars with a standard meal. Higher residue rate indicates the worse gastrointestinal transport function. The parameters include barium excretion rate after 48 hours, residue rate above rectosigmoid colon and residue rate within rectosigmoid colon. The barium excretion rate in normal people is more than or equal to 90%.

HRAM: HRAM is used to estimate the function of anal sphincter, rectal sensation, and anorectal reflex for diagnosing the defecation disorder constipation. The parameters include mean pressure of resting anal sphincter, maximum contraction pressure of resting anal sphincter, anal relaxation rate during analog defecation, first sensory value, defecation sensory value, and maximum tolerance.

BSFS: The Bristol stool score is used to assess fecal traits under the naked eye. 1 point: scattered hard lumps, like nuts; 2 points: sausage-like, but agglomerate; 3 points: sausage-like, but with cracks on the surface; 4 points: sausage-like or snake-like, smooth, and soft; 5 points: soft balls, clear edges; 6 points: unclear edges, mushy stools; 7 points: watery, no solids.

PAC-QOL: The quality of life assessment scale for patients with constipation includes 4 parts, including physiological, psychological, constipation's impact on life and treatment satisfaction, a total of 28 items, using Linker 5 grade. The higher the total score, the lower the quality of life, which means the more dissatisfied with the current state of life (see Supplementary Table 4).

PAC-SYM: The symptom self-assessment scale for patients with constipation includes 3 dimensions (abdominal symptoms, rectal symptoms, and stool symptoms) and 12 items. The Linker 5 scale is used. The higher the score, the more severe the constipation symptoms (see Supplementary Table 5).

F-SCFAs: Detection of short-chain fatty acids in feces contains acetic acid, propionic acid, butyric acid, isobutyric acid, valeric acid, isovaleric acid, and caproic acid. Feces samples were collected before and after the intervention and stored in the −80°C refrigerator in the biological sample bank of the China-Japan Friendship Hospital. Inspection and analysis after the test.

#### 2.11.4 Safety outcomes

Safety parameters include AEs and laboratory examinations (BR, LKF).

#### 2.11.5 DQ and compliance measures

After the test, the proportion of qualified diets and the frequency of various food intakes will be calculated. The diet intervention group should strictly follow the requirements of light vegetarian diet, while the nondiet intervention group does not need to observe the light vegetarian diet. Dietary compliance rate (DCR) means the ratio of qualified meals to total meals times 100. When DCR is greater than or equal to 80%, high compliance is achieved, while low DCR is less than 80%.

### 2.12 Adverse event

Adverse events refer to the negative effects caused by the intervention. During the trial, any abnormal reactions of the participants will be reported to the researchers. The time of onset, symptoms, degree, duration, and mitigation measures will be recorded in the CRF.

### 2.13 Data monitoring and confidentiality

Researchers will be trained in a unified way before the study to ensure it is performed correctly. Data will be locked in the airtight cabinet and will be entered and checked by double entry into the database established by data administrator using Excel 2019 edition (Microsoft Corporation, Redmond, WA) for management after completion of the study. An independent statistician will analyze the data while the procedure will be monitored by a third individual. The data of the patients involved in the trial will be confidential and will be used only by scientific research institutes. The publication or reporting of any research findings will not reveal the individual identity.

### 2.14 Planned statistical analysis

Continuous variables will be described as the mean ± standard deviation, median, or interquartile range. The number of cases, frequency, the rate, and the constituent ratio will be described by categorical variables. Variance analysis will be used for continuous variables in the baseline data having normal distribution and same variance. Wilcoxon rank sum test or Kruskal–Wallis H test will be used for the comparison of differences between groups that are not normally distributed or have uneven variance. Categorical variables will be tested by *χ*^2^ test or Fisher exact test. Wilcoxon rank sum test or Kruskal–Wallis H test will be used for the number of CSBM per week in primary outcome. *χ*^2^ test or Fisher exact test will be used for percent improvement of each symptom scale score in secondary outcome indicators. The result of 48-hour GITT will be analyzed using *χ*^2^ or Fisher test. Wilcoxon rank sum test or Kruskal–Wallis H test will be used for measurement data in HRAM, while *χ*^2^ test or Fisher exact test will be used for counting data. Safety, dietary quality, compliance indicators will be tested by *χ*^2^ test or Fisher exact test. Complying with the intent-to-treat and per-protocol principle, all data of the patients randomized and at least 1 assessment (include baseline assessment) will be analyzed. For dropouts and terminations, their missing data will be obtained by last-observation-carried-forward method. All statistical analyses will be performed by SPSS23 software (IBM, Chicago, IL), and *P* value <.5 will be considered statistically significant.

### 2.15 Ethics and dissemination

This study has been approved by China-Japan Friendship Hospital clinical research ethics committee (No. 2017-46-1). Participants will need to sign the informed consent before the entering the study. During the intervention, patients have the right to withdraw at any condition and will not affect subsequent treatment (personal treatments from the expert team). We must submit a written application to the ethics committee if the protocol needs to be modified. The committee members will decide whether to modify it. The results of the study will be sent to researchers, participants, and disseminated to the public through academic conferences and journals. This is an open access article, which permits unrestricted use when the original work is correctly cited.

## 3 Discussion

FC is a common disease of the digestive system and is characterized by persistent or recurrent difficulty in defecation. Constipation can cause digestive symptoms such as loss of appetite, abdominal distension, hemorrhoids, and colon polyps. It can also lead to cardiovascular and cerebrovascular diseases,^[18–20]^ kidney diseases,^[21,22]^ and even sudden death. At present, traditional Chinese and Western medicines used to treat functional constipation can only temporarily improve constipation. Constipation can easily recur after stopping the medication. Some traditional Chinese medicines also cause damage to the intestinal tract (melanosis coli).^[23,24]^ Long-term improvement of constipation can be achieved through the management of patients’ cognitive behavioral patterns. Studies have shown that dietary fiber supplementation can improve chronic constipation, but there is no intervention study on constipation with a vegan diet.^[25–27]^ In clinical practice, maintenance of FC improvement has been observed in patients who persist in LVD for a period of time without any drug use. TCM is a national medicine in China.^[28,29]^ It has been caring for the health of Chinese people from ancient times to present. TCM has mature experience in the treatment of constipation. Qing Jiang Tiao Chang Fang is a safe and effective prescription. However, these are only the results of clinical observations and need to be scientifically validated in clinical trials. This RCT aims to clarify the efficacy and safety of LVD and QJTCF on FC by designing rigorous trials. The results of the study can provide more evidence for dietary intervention and TCM intervention on FC, and to some extent, clarify the complementary effect of diet on drugs. There are still some limitations in this study:

1. Dietary intervention is a cognitive behavioral therapy, and there are many objective factors that will hinder the practice of light vegetarian diet and affect the experimental results.
2. The experiment explored the metabolic effects of vegetarian diet and traditional Chinese medicine on fecal short-chain fatty acids, but no other metabolites were studied.
3. The current study belongs to exploratory study with small sample size.

Therefore, based on this study, a large sample size, multicenter RCT should be designed in the future to verify the effectiveness and safety of LVD and QJTCF on FC. In terms of mechanism, if intestinal flora, metabolism, power, and other angles can be integrated to study, it can be more convincing.

## Acknowledgment

The authors thank the doctors and other staff in the Department of Gastroenterology and Gastroenterology of Chinese Medicine of China-Japan Friendship Hospital for their assistance. LX is the first author. The authors thank MB of the Pharmacy Department of China-Japan Friendship Hospital for assisting in the packaging of drugs and placebos. They thank Sun Ruihua, Clinical Research Institute of China-Japan Friendship Hospital, for providing statistical guidance.

## Author contributions

**Conceptualization:** Xinyuan Liu, Yu Liu, Shukun Yao.

**Data curation:** Xinyuan Liu, Yu Liu, Jialiang Chen, Huijing Wang.

**Formal analysis:** Xinyuan Liu.

**Funding acquisition:** Shukun Yao.

**Investigation:** Yuehua Ding, Yuanchen Zhou, Xinyuan Liu, Huijing Wang, Qianqian Wang, Zuohu Niu, Zhangjun Yun.

**Methodology:** Xinyuan Liu, Yu Liu, Jialiang Chen, Qianqian Wang, Bingzhi Ma, Shukun Yao.

**Project administration:** Xinyuan Liu, Shukun Yao.

**Resources:** Shukun Yao.

**Software:** Xinyuan Liu, Jialiang Chen, Shukun Yao, Zuohu Niu, Zhangjun Yun.

**Supervision:** Shukun Yao.

**Visualization:** Xinyuan Liu, Bingzhi Ma.

**Writing – original draft:** Xinyuan Liu, Yu Liu

**Writing – review & editing:** Xinyuan Liu, Shukun Yao.

## Supplementary Material

Supplemental Digital Content

## Supplementary Material

Supplemental Digital Content

## Supplementary Material

Supplemental Digital Content

## Supplementary Material

Supplemental Digital Content

## Supplementary Material

Supplemental Digital Content
