# Supplementary material for: Effectiveness and safety of light vegetarian diet and Qingjiang Tiaochang Recipe for functional constipation: An exploratory study protocol for randomized controlled trial
Source: Medicine (Baltimore). 2020 Sep 25;99(39):e21363. doi: 10.1097/MD.0000000000021363 (PMC7523849; doi:10.1097/MD.0000000000021363)
Supplement: Supplemental Digital Content [file medi-99-e21363-s001.docx]

| Table 3 Dietary Questionnaire | | | | | | | | | | | |
| --- | --- | --- | --- | --- | --- | --- | --- | --- | --- | --- | --- |
| **Dietetic Varieties** | **The food name(Semi–quantitative per time)** | **Average frequency of consumption over the past year** | | | | | | | | | |
|  |  | never | Less than 1 time a month | 1 to 3 times a month | 1 time per week | 2 to 4 times a week | 5 to 6 times a week | 1 time a day | 2 to 3 times a day | 4 to 5 times a day | More than 6 times a day |
| Spicy and stimulating food | chilli（no spicy, less spicy, medium spicy, very spicy） |  |  |  |  |  |  |  |  |  |  |
|  | Sichuan peper (no, a little, Medium quantity, large quantity) |  |  |  |  |  |  |  |  |  |  |
|  | Peper (no, a little, Medium quantity, large quantity) |  |  |  |  |  |  |  |  |  |  |
|  | Green onion (no, a little, Medium quantity, large quantity) |  |  |  |  |  |  |  |  |  |  |
|  | Garlic (1 clove) |  |  |  |  |  |  |  |  |  |  |
|  | Leek (50g) |  |  |  |  |  |  |  |  |  |  |
|  | Toona sinensis (50g) |  |  |  |  |  |  |  |  |  |  |
|  | Allium bulbus (50g) |  |  |  |  |  |  |  |  |  |  |
|  | Onion (50g) |  |  |  |  |  |  |  |  |  |  |
| Fried food | Deep–Fried Dough Sticks (1 root) |  |  |  |  |  |  |  |  |  |  |
|  | Oil cake (1 piece) |  |  |  |  |  |  |  |  |  |  |
|  | Fries (75g) |  |  |  |  |  |  |  |  |  |  |
|  | Fried peanuts (50g) |  |  |  |  |  |  |  |  |  |  |
|  | Other fried food (50g) |  |  |  |  |  |  |  |  |  |  |
| Barbecue food | Kabob (1 kebab) |  |  |  |  |  |  |  |  |  |  |
|  | Roast vegetarian food (1bunch) |  |  |  |  |  |  |  |  |  |  |
| Stir–fried food | Roasted peanuts (50g) |  |  |  |  |  |  |  |  |  |  |
|  | Fried melon seeds (50g) |  |  |  |  |  |  |  |  |  |  |
|  | Other stir–fried food (50g) |  |  |  |  |  |  |  |  |  |  |
| Meat | Beef or mutton(50g) |  |  |  |  |  |  |  |  |  |  |
|  | Pork(50) |  |  |  |  |  |  |  |  |  |  |
|  | Fish(50g) |  |  |  |  |  |  |  |  |  |  |
|  | Shrimp or other seafood(50g) |  |  |  |  |  |  |  |  |  |  |
|  | Chicken, duck or other poultry (50g) |  |  |  |  |  |  |  |  |  |  |
| Eggs | egg（1 egg） |  |  |  |  |  |  |  |  |  |  |
| Milk | Liquid milk(250ml) |  |  |  |  |  |  |  |  |  |  |
|  | Solid milk(50g) |  |  |  |  |  |  |  |  |  |  |
| Coffee | Coffee(1 cup) |  |  |  |  |  |  |  |  |  |  |
| Tea | Tea(10g) |  |  |  |  |  |  |  |  |  |  |
| Water | Water(500ml) |  |  |  |  |  |  |  |  |  |  |
| Vegetables | Leafy vegetables (50g) |  |  |  |  |  |  |  |  |  |  |
|  | Fruit vegetables (50g) |  |  |  |  |  |  |  |  |  |  |
|  | Rhizome vegetables (50g) |  |  |  |  |  |  |  |  |  |  |
| Fruits | Fruits (500g) |  |  |  |  |  |  |  |  |  |  |
| Staple food | - [cooked](javascript:void(0);) [wheaten](javascript:void(0);) [food](javascript:void(0);)(50g) |  |  |  |  |  |  |  |  |  |  |
|  | Rice food (50g) |  |  |  |  |  |  |  |  |  |  |
|  | Cereals, Whole grains, semen coicis (50g) |  |  |  |  |  |  |  |  |  |  |
|  | Beans (50g) |  |  |  |  |  |  |  |  |  |  |
| Liquor | Spirits (50ml) |  |  |  |  |  |  |  |  |  |  |
|  | red wine (50ml) |  |  |  |  |  |  |  |  |  |  |
|  | Beer (50ml) |  |  |  |  |  |  |  |  |  |  |
|  | Other alcoholic drinks (50ml) |  |  |  |  |  |  |  |  |  |  |
| Regular diet (regular and quantitative) | |  |  |  |  |  |  |  |  |  |  |

Effectiveness and safety of light vegetarian diet and Qingjiang Tiaochang Recipe for functional constipation : An exploratory study protocol for randomized controlled trial , Liu Xinyuan
