# Supplementary material for: Effectiveness and safety of light vegetarian diet and Qingjiang Tiaochang Recipe for functional constipation: An exploratory study protocol for randomized controlled trial
Source: Medicine (Baltimore). 2020 Sep 25;99(39):e21363. doi: 10.1097/MD.0000000000021363 (PMC7523849; doi:10.1097/MD.0000000000021363)
Supplement: Supplemental Digital Content [file medi-99-e21363-s002.docx]

Table 4：Constipation–related Symptoms Rating Scale

| **Degree**: visual analogue scale was used.  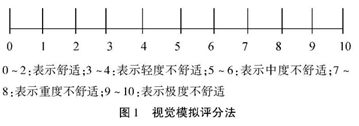  0: no discomfort; 10: extreme discomfort.  Patients will give a score of 0–10 according to their actual feelings.  **Frequency**: average weekly frequency of such discomfort. | | | |
| --- | --- | --- | --- |
| **symptoms** | **scores** | | |
|  | **Degree(scores)** | **Frequency(times/week)** | **Total scores（degree*frequency/bowel movements per week）** |
| **1.sense of straining** |  |  |  |
| **2.imcomplete evacuation** |  |  |  |
| **3.manual maneuvers to facilitate** |  |  |  |
| **4.no defecation sensation** |  |  |  |
| **5.sensation of anorectal obstruction** |  |  |  |
| **6.burning sensation of anus** |  |  |  |
| **7.anus droop sensation** |  |  |  |

Effectiveness and safety of light vegetarian diet and Qingjiang Tiaochang Recipe for functional constipation : An exploratory study protocol for randomized controlled trial , Liu Xinyuan
