# Supplementary material for: Effectiveness and safety of light vegetarian diet and Qingjiang Tiaochang Recipe for functional constipation: An exploratory study protocol for randomized controlled trial
Source: Medicine (Baltimore). 2020 Sep 25;99(39):e21363. doi: 10.1097/MD.0000000000021363 (PMC7523849; doi:10.1097/MD.0000000000021363)
Supplement: Supplemental Digital Content [file medi-99-e21363-s003.docx]

Table 5 TCM Syndrome Scale

| symptoms | | degree | grade | scores |
| --- | --- | --- | --- | --- |
| abdominal distention | | no discomfort | 0 normal |  |
|  |  | slightly distention at sometimes without affecting work and rest | 1 mild |  |
|  |  | obvious but tolerable distention occasionally affecting work and rest | 2 medium |  |
|  |  | persistent unbearable distention with drugs to alleviate | 3 severe |  |
| decrease in diet | | no decrease | 0 normal |  |
|  |  | 1/4 reduction in food intake | 1 mild |  |
|  |  | 1/3 reduction in food intake | 2 medium |  |
|  |  | more than 1/2 reduction in food intake | 3 severe |  |
| fatigue and weakness | | no discomfort | 0 normal |  |
|  |  | limb burnout and light physical work | 1 mild |  |
|  |  | fatigue limbs and reluctant to adhere to daily activities | 2 medium |  |
|  |  | Weakness and unwillingness to move all day | 3 severe |  |
| belch | | no discomfort | 0 normal |  |
|  |  | occasionally belch | 1 mild |  |
|  |  | sometimes belch | 2 medium |  |
|  |  | frequent belching and aggravation after eating even acid reflux | 3 severe |  |
| nausea and vomiting | | no discomfort | 0 normal |  |
|  |  | occasionally nausea | 1 mild |  |
|  |  | sometimes nausea and occasional vomiting | 2 medium |  |
|  |  | frequent nausea and sometimes vomiting | 3 severe |  |
| sticky stool | | no discomfort | 0 normal |  |
|  |  | occasionally sticky stool | 1 mild |  |
|  |  | sometimes sticky stool | 2 medium |  |
|  |  | frequent sticky stool | 3 severe |  |
| difficult defecation | | no discomfort | 0 normal |  |
|  |  | light difficult defecation | 1 mild |  |
|  |  | poor stool and difficulty in defecation | 2 medium |  |
|  |  | difficult defecation and need to take laxatives | 3 severe |  |
| dry stool | | no dry stool | 0 normal |  |
|  |  | occasionally dry stool | 1 mild |  |
|  |  | sometimes dry stool | 2 medium |  |
|  |  | frequent dry stool | 3 severe |  |
| tongue manifestation | 0 | normal | |  |
|  | 1 | pale tongue□ red tongue□ dark tongue□ fatty tongue□ tooth trace□ others□→description_____ | |  |
|  |  | white and thick coat□ thin and yellow coat□ yellow and greasy coat□ yellow and thick coat□ less coat□ others□ description____ | |  |
| type of pulse | 0 | normal | |  |
|  | 1 | deep pulse □slippery pulse □ string pulse □ thready pulse□  others□ description_______________________________ | |  |

Effectiveness and safety of light vegetarian diet and Qingjiang Tiaochang Recipe for functional constipation : An exploratory study protocol for randomized controlled trial , Liu Xinyuan
