# Supplementary material for: Effectiveness and safety of light vegetarian diet and Qingjiang Tiaochang Recipe for functional constipation: An exploratory study protocol for randomized controlled trial
Source: Medicine (Baltimore). 2020 Sep 25;99(39):e21363. doi: 10.1097/MD.0000000000021363 (PMC7523849; doi:10.1097/MD.0000000000021363)
Supplement: Supplemental Digital Content [file medi-99-e21363-s004.docx]

**PAC-QOL ( PATIENT ASSESSMENT OF CONSTIPATION )**

The following questions are designed to measure the impact constipation has had on your daily life during the past 2 weeks. For each question, please tick one box.

| The following questions ask you about the intensity of your symptoms. To what extent, during the past 2 weeks… | Not at all  0 | A little bit  1 | Moderately  2 | Quite a bit  3 | Extremely  4 |
| --- | --- | --- | --- | --- | --- |
| 1. have you felt bloated to the point of bursting? | □ | □ | □ | □ | □ |
| 2. have you felt heavy because of your constipation? | □ | □ | □ | □ | □ |

| The next few questions ask you about the effects of constipation on your daily life. How much of the time, during the past 2 weeks… | None of the time  0 | A little of the time  1 | Some of the time  2 | Most of the time  3 | All of the time  4 |
| --- | --- | --- | --- | --- | --- |
| 3. have you felt any physical discomfort? | □ | □ | □ | □ | □ |
| 4. have you felt the need to open your bowel but not been able to? | □ | □ | □ | □ | □ |
| 5. have you been embarrassed to be with other people? | □ | □ | □ | □ | □ |
| 6. have you been eating less and less because of not being able to have bowel movements? | □ | □ | □ | □ | □ |

| The next few questions ask you about the effects of constipation on your daily life. How much of the time, during the past 2 weeks… | Not at all  0 | A little bit  1 | Moderately  2 | Quite a bit  3 | Extremely  4 |
| --- | --- | --- | --- | --- | --- |
| 7. have you had be careful about what you eat? | □ | □ | □ | □ | □ |
| 8. have you had a decreased appetite? | □ | □ | □ | □ | □ |
| 9. have you been worried about not been able to choose what you eat(for example, at friend’s)? | □ | □ | □ | □ | □ |
| 10. have you been embarrassed about staying in the toilet for so long when you were away from home? | □ | □ | □ | □ | □ |
| 11. have you been embarrassed about having to go to toilet so often when you were away from home? | □ | □ | □ | □ | □ |
| 12. have you been worried about having to change your daily routine (for example, travelling, being away from home)? | □ | □ | □ | □ | □ |

| The next few questions ask your feelings. How much of the time, during the past 2 weeks… | None of the time  0 | A little of the time  1 | Some of the time  2 | Most of the time  3 | All of the time  4 |
| --- | --- | --- | --- | --- | --- |
| 13. have you felt irritable because of your condition? | □ | □ | □ | □ | □ |
| 14. have you been upset by your condition? | □ | □ | □ | □ | □ |
| 15. have you felt obsessed by your condition? | □ | □ | □ | □ | □ |
| 16. have you felt stressed by your condition? | □ | □ | □ | □ | □ |
| 17.have you been less self-confident because of your condition? | □ | □ | □ | □ | □ |
| 18. have you felt in control of your situation? | □ | □ | □ | □ | □ |

| The next few questions ask you about your feelings. To what extent, during the past 2 weeks… | Not at all  0 | A little bit  1 | Moderately  2 | Quite a bit  3 | Extremely  4 |
| --- | --- | --- | --- | --- | --- |
| 19. have you been worried about not knowing when you are going to be able to open your bowels? | □ | □ | □ | □ | □ |
| 20. have you been worried about not being able to open your bowels when you needed to? | □ | □ | □ | □ | □ |
| 21. have you been more and more bothered by not being able to open your bowels? | □ | □ | □ | □ | □ |

| The next few questions ask about your life with constipation. How much of the time, during the past 2 weeks… | None of the time  0 | A little of the time  1 | Some of the time  2 | Most of the time  3 | All of the time  4 |
| --- | --- | --- | --- | --- | --- |
| 22. have you been afraid that your condition will get worse? | □ | □ | □ | □ | □ |
| 23. have you felt that your body was not working properly? | □ | □ | □ | □ | □ |
| 24. have you had fewer bowel movements than you would like? | □ | □ | □ | □ | □ |

| The next few questions ask you about how satisfied you are. To what extent, during the past 2 weeks… | Not at all  0 | A little bit  1 | Moderately  2 | Quite a bit  3 | Extremely  4 |
| --- | --- | --- | --- | --- | --- |
| 25. have you been satisfied with how often you open your bowels? | □ | □ | □ | □ | □ |
| 26. have you been satisfied with the regularity with which you open your bowels? | □ | □ | □ | □ | □ |
| 27. have you been satisfied with your bowel function? | □ | □ | □ | □ | □ |
| 28. have you been satisfied with your treatment | □ | □ | □ | □ | □ |

Effectiveness and safety of light vegetarian diet and Qingjiang Tiaochang Recipe for functional constipation : An exploratory study protocol for randomized controlled trial , Liu Xinyuan
