# Supplementary material for: Effectiveness and safety of light vegetarian diet and Qingjiang Tiaochang Recipe for functional constipation: An exploratory study protocol for randomized controlled trial
Source: Medicine (Baltimore). 2020 Sep 25;99(39):e21363. doi: 10.1097/MD.0000000000021363 (PMC7523849; doi:10.1097/MD.0000000000021363)
Supplement: Supplemental Digital Content [file medi-99-e21363-s005.docx]

| How severe have each of these symptoms been in the past 2 weeks? | Absent  0 | Mild  1 | Moderate  2 | Severe  3 | Very severe  4 |
| --- | --- | --- | --- | --- | --- |
| 1. Discomfort in your stomach |  |  |  |  |  |
| 1. Pain in your stomach |  |  |  |  |  |
| 1. Bloating in your stomach |  |  |  |  |  |
| 1. Stomach cramps |  |  |  |  |  |
| 1. Painful bowel movements |  |  |  |  |  |
| 1. Rectal burning or tearing during or after a bowel movement |  |  |  |  |  |
| 1. Rectal bleeding or tearing during or after a bowel movement |  |  |  |  |  |
| 1. Incomplete bowel movement, as though you didn’t “finish” |  |  |  |  |  |
| 1. Stools that were too hard |  |  |  |  |  |
| 1. Stools that were too small |  |  |  |  |  |
| 1. Straining or squeezing to try to pass stools |  |  |  |  |  |
| 1. Feeling like you had pass a stool but you couldn’t (false alarm) |  |  |  |  |  |

**PAC-SYM (PATIENTS ASSESSMENT OF CONSTIPATION)**

This questionnaire asks you about your in the past 2 weeks. Answer every question according to your symptoms, as accurately as possible. There are no right or wrong answer.

For each of the following symptoms, please indicate how severe your symptoms have been during the past two weeks. If you have no symptoms in the past two weeks, tick 0. If the symptoms look like mild, tick 1. If symptoms seemed moderate, Tik 2. If the symptoms look severe, tick 3. If the symptoms look like very severe, tick 4. Please answer every question.

Effectiveness and safety of light vegetarian diet and Qingjiang Tiaochang Recipe for functional constipation : An exploratory study protocol for randomized controlled trial , Liu Xinyuan
